# Exploring the impact on emotional wellbeing of having a spouse or cohabiting partner with elevated Problem Gambling Severity Index (PGSI) scores: Secondary analysis of cross‐sectional health survey data

**DOI:** 10.1111/add.70154

**Published:** 2025-09-03

**Authors:** Sarah Tipping, Heather Wardle, Robert Pryce

**Affiliations:** ^1^ University of Glasgow Glasgow UK; ^2^ University of Sheffield Sheffield UK

**Keywords:** gambling, harms, affected others, PGSI, emotional wellbeing, mental health, concerned significant others, spouses

## Abstract

**Background/Aims:**

To conduct an exploratory analysis of the association between the mental health and emotional wellbeing of an individual and the Problem Gambling Severity Index (PGSI) scores of their spouse or cohabiting partner.

**Design:**

Observational study using two sources of secondary data: the Health Survey for England (HSE) and the Scottish Health Survey (SHeS). Data from 2012, 2015, 2016, 2017 (SHeS only) and 2018 (HSE only) were combined to create a single data set. The data contained household identifiers and a household grid, allowing cohabiting couples to be identified.

**Setting:**

England and Scotland.

**Participants:**

20 752 individuals (in 10 376 couples) where both partners had completed the PGSI.

**Measurements:**

Outcome measures were four self‐reported measures of emotional wellbeing: a binary measure indicating a long‐term mental health condition, a scale question on life satisfaction, the twelve‐item General Health Questionnaire (GHQ‐12) and the Warwick Edinburgh Mental Health and Wellbeing Scale (WEMWBS). Gambling harms were measured using the PGSI. Controls included socio‐demographic/economic characteristics, and a binary variable indicating whether the individual had spent money in the last 12 months on gambling.

**Findings:**

Regression models showed a statistically significant association between lower emotional wellbeing, measured by WEMWBS [Coef. 0.022; 95% confidence interval (CI) = 0.004–0.040], GHQ‐12 Likert scale (Coef. 0.021; 95% CI = 0.000–0.043) and life satisfaction (Coef. 0.036; 95% CI = 0.005–0.067), among individuals who themselves had a PGSI score of zero but whose partner had elevated PGSI scores, when controlling for the individual's own gambling participation and other socio‐demographic household and individual characteristics. There was no evidence of an effect between partner's PGSI score and an increased likelihood of a long‐term mental health condition amongst the same group.

**Conclusions:**

Decrements to an individual's emotional wellbeing appear to be strongly associated with increased Problem Gambling Severity Index (PGSI) scores among their spouse/cohabiting partner, with an individual's emotional wellbeing declining as their partner's PGSI score increases.

## INTRODUCTION

Gambling harms are the negative consequences of gambling, impacting not only on the individual who gambles but also their close contacts, community and the wider population. These harms impact on a range of connected areas, including finances, health, emotional wellbeing, personal relationships, work and study, and can lead to criminal activity and suicidality [1].

For every person experiencing problem gambling it is estimated that an average of six others are indirectly affected, such as partners, children, parents, friends or colleagues [2]. These close contacts experience a wide range of harms, as evidenced by a growing international body of work. Several studies have found close contacts of those experiencing problem gambling are more likely to experience relationship conflict, financial problems, poorer physical and mental wellbeing, emotional distress and higher levels of risky alcohol use, associated with their close contact’s gambling [3, 4, 5, 6]. These harms include physical and mental health problems linked to sustained mental distress [7, 8, 9]. Harms have been shown to compound each other in damaging ways [10, 11]. Negative impacts can be ongoing and can remain a year on [12].

The type of relationship between those gambling and others has consequences for the risk of harm. Those living in the closest proximity to the individual affected by problem gambling, who are closer emotionally, financially and physically, experience the greatest harms [13, 14]. Partners and ex‐partners of those who experience problem gambling are more likely to report stress‐related health problems than other close contacts [15, 16]. It is often the spouse or cohabiting partner of the individual experiencing disordered gambling who is most likely to seek support [17].

To date, much of the evidence has focused on the impact of problem or disordered gambling on close contacts. The evidence base considering how those experiencing lower level harms might also impact on those close to them is nascent. However, there is a wider acceptance that harms do not only impact a small number of disordered individuals but also impact those who are sub‐threshold for the experience of gambling disorder [6], with gambling harms viewed as existing upon a spectrum of risk severity [18]. Evidence suggests that those from sub‐threshold groups contribute a greater burden of harms than those experiencing gambling disorder because of the larger population numbers [19, 20]. To date, only a handful of studies have examined the health and wellbeing of close contacts of people who gambled across the spectrum of risk. In 2023, Tulloch *et al*. found financial harms occurred for close contacts of those experiencing both moderate‐ and high‐risk gambling, whilst lower wellbeing, social harms and poorer health were more strongly associated with close contacts of those experiencing higher gambling harms [21]. A qualitative study looking at the impact of harms on close contacts also showed that harms could occur across the risk spectrum, also showing that the impact of a close contact’s gambling varied by the closeness of the relationship, the inter‐dependence of the two people’s lives and the close contact’s level of social support [22]. Our objectives were to conduct exploratory secondary analysis of English and Scottish data to identify whether there were detriments to the emotional health of people whose spouses and cohabiting partners have elevated Problem Gambling Severity Index (PGSI) scores, investigating the impact across the full PGSI spectrum.

## METHODS

### Design

The Health Survey for England (HSE) [23] and Scottish Health Survey (SHeS) [24] are both annual, large‐scale, random probability surveys designed to monitor trends in their respective nation’s health. All adults residing within the household are eligible to take part. Both studies include a self‐completion element where participants aged 16 years and over are asked a series of more sensitive questions, including self‐reported mental health, emotional wellbeing and gambling, including the PGSI.

The HSE and SHeS data from 2012, 2015, 2016, 2017 (SHeS only) and 2018 (HSE only) were combined (each year of data appended) to create a single data set of individuals aged 16 years or over that covered both England and Scotland (*n* = 50 346). The HSE data for 2015, 2016 and 2018 were obtained under special licence to enable household identifiers to be accessed. The other survey year data sets already include household indicators within their standard access arrangements. In addition to household identifiers, the data included information on the relationships of each household member to all others. The combined data set was weighted to be representative of adults aged 16 years and over living in England and Scotland (for details, see Appendix S1).

These data were used to identify households containing married or cohabiting couples where both partners had participated in the survey and completed the PGSI. These data contained 20 752 individuals in 10 376 couples. From these, 20 091 individuals were selected for analysis. These were individuals with a PGSI score of zero but whose partner’s PGSI scores ranged from 0 to 27. The remaining cases (661 individuals whose own PGSI score was greater than zero) were excluded from the analysis. This was done to reduce the risk of the individual’s own PGSI score confounding the results when examining the impact of the partner’s PGSI on the individual’s wellbeing.

It should be noted that there is correlation between the PGSI scores of individuals and the PGSI scores of their spouses and cohabiting partners. The 661 individuals that were excluded had partners whose PGSI scores were also higher; the mean partner PGSI score of this group was 0.366 (SD = 1.487). Analysis of this group was deemed outside the scope of this specific article but is noted for further investigation. A decision was made to focus on individuals whose own PGSI was zero.

### Outcomes

The outcome measures used in the study were four self‐reported measures of mental health and wellbeing:

1. A dichotomous variable indicating the presence of a long‐term mental health condition (5.13%, 95% CI = 4.75%–5.54%). Participants were asked whether they had any physical or mental health conditions or illness that had lasted or was expected to last 12 months or more, and if so to specify up to six conditions. Responses were used to generate a dichotomous variable. This measure was included on both HSE and SHeS for all survey years.
2. The 14‐item Warwick–Edinburgh Mental Wellbeing Scale (WEMWBS), an indicator of probable depression, was included within the self‐completion questionnaire. This validated scale [25] asks participants 14 questions about their feelings and thoughts over the previous 2 weeks, with responses scored from 1 to 5. These scores are then combined to create an overall scale, ranging from 14 to 70 (mean = 51.98, SD = 8.21). Lower scores reflect a higher likelihood of experiencing depression. WEMWBS was not available for HSE 2018.
3. The 12‐item General Health Questionnaire (GHQ‐12) is a validated measure of emotional distress [26]. Included within the self‐completion questionnaire, the GHQ‐12 comprises 12 questions that ask participants how often over the previous 4 weeks they have experienced behaviours that may be symptoms of mental distress. Responses to each of the 12 items were given a score between 0 and 3, where ‘More so than usual’ = 0, ‘About the same as usual’ = 1, ‘Less so than usual’ = 2 and ‘Much less than usual’ = 3. These scores were then summed to give an overall score ranging from 0 to 36 (mean = 10.65, SD = 4.73). This scoring approach has been used widely, giving a wider and smoother scoring approach appropriate for population‐based analyses. GHQ‐12 was not included in HSE 2015.
4. A measure of life satisfaction was included in the SHeS main questionnaire and the HSE self‐completion questionnaire. Participants were asked to rate their current overall satisfaction with life on a scale of 0 to 10, where 0 is ‘not at all satisfied’ and 10 is ‘completely satisfied’ (mean = 7.75, SD = 1.79). The life satisfaction score was not included in HSE 2012 or 2015.

Where data were not available for a survey year, that survey year was excluded from the analysis.

### Exposure

Gambling harms for both the participant and their partner were measured using the PGSI [27]. This a validated tool for the identification of gambling harms that was asked of anyone who had gambled in the past year within the self‐completion questionnaire. The PGSI comprises nine questions that are combined to produce a score ranging from 0 to 27. All participants have PGSI = 0; the mean PGSI for their partners is 0.088 (SD = 0.794, *α* = 0.91).

### Controls

The individual’s gambling participation and a range of socio‐demographic characteristics were included as control variables. Gambling participation was measured as whether the individual had spent money on gambling in the past 12 months (60.77%, 95% CI = 59.82%–61.72%).

The remaining control measures were sex, age (grouped into 10‐year bands), ethnicity (coded as white or other, because of the small base sizes), religion (coded as no religion, Christian or other), the individual’s weekly alcohol consumption [non‐drinker; moderate (men, <22 units/women, <15 units); hazardous (men, 22–50 units/women, 15–35 units); harmful (men, 50+ units/women, 35+ units)], smoking status [never; ex‐smoker (occasional); ex‐smoker (regular); current smoker], passive smoking exposure (yes/no), the individual’s economic activity (full/part‐time employment; education; training), their highest educational qualification (degree or higher; A‐levels or equivalent; GCSEs or equivalent; other; none), their National Statistics Socio‐economic Classification (NS‐SEC, five groups, missing data are included as a separate category), equivalised household income (five groups based on quintiles, with missing data included as a separate category), tenure, number of cars in the household, whether the couple were married or cohabiting and household type (based on household size and presence of children aged 0–15 years in the household). Local deprivation was measured using English and Scottish Indices of Multiple Deprivation (IMD) scores, matched at the ‘Output Area’ and quintiled for analysis; urbanicity was measured using the Office for National Statistics (ONS) urban–rural classification and grouped into urban/other. Region of residence was based on government region, with Scotland included as a separate region. Finally, survey year was also included as a control variable. Details about missing values are given in Appendix S2.

### Analyses

The estimates presented in all tables are based on weighted data with true (unweighted) bases included. All analyses were carried out in Stata 18 (StataCorp LLC, College Station, TX, USA) using the ‘svy’ suite of commands to account for weights, stratification and the clustering of responses within couples. The cluster variable was an anonymised household indicator, the stratification variable was based on the main regional stratifier used in each survey (Strategic Health Area/Region in HSE; Scottish Health Board in SHeS), split by survey year. More details are available in Appendix S1.

Bivariate descriptive statistics were used to review the demographic characteristics of the 20 091 sample members, comparing mean partner PGSI scores across different characteristics. This standardised set of characteristics were used as control variables in the full models.

Correlation coefficients and scatter plots were used to look at the bivariate associations between the individual’s emotional wellbeing scores and their partner’s PGSI score. The scatter plots are overlaid with a linear fitted line to show the underlying trend. Mean partner PGSI is shown for individuals with and without a long‐term mental health condition, and this is also shown as a bar chart.

The impact of increasing partner PGSI score on an individual’s emotional wellbeing was explored using a series of regression models. Each regression model used a different health measure as an outcome and the partner’s continuous PGSI scores as a predictor. Different regression models were required for different wellbeing measures. The presence of a long‐term mental health condition was a binary outcome and hence a logistic regression model was used, with the results reported as odds ratios (ORs). The three scale measures of emotional wellbeing – WEMWBS, Likert GHQ‐12 and life satisfaction – were modelled using linear regression, with coefficients reported.

To aid interpretation, both the WEMWBS and the life satisfaction scores were reverse‐coded so that a higher score indicated lower levels of emotional wellbeing or life satisfaction. All three wellbeing variables were standardised to give a mean of zero and standard deviation of one, again, to aid interpretation. Partner PGSI was entered into each regression as a continuous variable, rather than a banded variable, as the aim was to treat PGSI as a continuum.

As the regressions were run on the sub‐sample of individuals who had a PGSI score of zero, they investigate the relationship between partner PGSI and mental health and wellbeing outcomes for individuals who themselves are deemed not to be at risk from gambling harms. Two regressions were run for each outcome to get unadjusted and adjusted results. The second regression model contained a standardised set of controls.

The diagnostics for each model were checked. Those for the GHQ‐12 linear regression model suggested the normality assumption did not fully hold, suggesting a different approach to the modelling may have been more suitable. To test this, the GHQ‐12 analysis was repeated using Poisson regression and using the original, unstandardised GHQ‐12 Likert scale as the outcome variable. More details are given in Appendix S3. The results corroborate the associations indicated by the linear regression model presented in this paper and are given in Appendix S3: Table S3.1. Additionally, a set of E‐values were generated to explore the strength of existing associations against unmeasured confounders, these are described in Appendix S4 and presented in Tables S4.1 and S4.2. A full STROBE checklist is given in Appendix S5.

### Ethics

This study was approved by the University of Glasgow Research Ethics System (application number 400230254; project title ‘Exploring gambling harms: secondary analysis of survey data’; College of Social Sciences committee).

## RESULTS

Table 1 presents the socio‐demographic characteristics of the individuals (*n* = 20 091). Individuals who were female, younger, cohabiting rather than married, had children aged 0–15 years in the household and lived in in the most deprived area quintile had partners with higher mean PGSI scores.

**TABLE 1:** Characteristics of the sample.

Individual’s characteristics	Mean Partner PGSI	Standard deviation of partner PGSI	Weighted proportion of individuals (%)	Lower 95% CI for proportion of individuals (%)	Upper 95% CI for proportion of individuals (%)	Unweighted base
Age group						
16–34 years	0.183	1.188	21.22	20.26	22.22	3421
35–64 years	0.080	0.732	56.86	55.78	57.94	11 347
65+ years	0.019	0.325	21.92	21.08	22.78	5323
Sex						
Male	0.033	0.485	52.55	52.33	52.76	9866
Female	0.149	1.030	47.45	47.24	47.67	10 225
Ethnicity (grouped)						
Other	0.087	0.920	10.16	9.49	10.88	1448
White	0.089	0.778	89.84	89.12	90.51	18 643
Religion (grouped)						
No religion	0.108	0.867	34.22	33.27	35.19	7004
Christian – Catholic	0.087	0.736	18.68	17.93	19.44	3357
Christian – all other denominations	0.073	0.718	39.07	38.14	40.02	8605
Any other religion	0.079	0.936	8.03	7.41	8.69	1125
Economic activity						
In employment, self‐employed or government training	0.098	0.821	64.24	63.29	65.18	11 976
In full‐time education	0.107	0.684	1.13	0.94	1.36	246
Retired	0.025	0.375	23.06	22.21	23.92	5584
International Labour Organization (ILO) unemployed	0.175	1.035	2.00	1.75	2.29	367
Other inactive	0.157	1.195	9.57	9.06	10.09	1918
Highest educational qualification						
Degree (or equivalent) or higher	0.081	0.756	33.54	32.58	34.5	6576
Higher education below degree	0.081	0.749	12.23	11.69	12.79	2450
A‐Levels/Scottish Highers/or equivalent	0.106	0.919	15.24	14.6	15.9	2953
GCSEs/Scottish Standard Grades/or equivalent	0.093	0.725	21.89	21.15	22.64	4492
Other	0.110	0.899	1.12	0.97	1.29	237
No qualifications	0.085	0.856	16.00	15.3	16.72	3383
Individual National Statistics Socio‐economic Classification (NS‐SEC)						
Managerial and professional occupations	0.075	0.722	41.85	40.92	42.79	8269
Intermediate occupations	0.107	0.782	14.12	13.56	14.71	2837
Small employers and own account workers	0.071	0.774	10.35	9.8	10.92	1981
Lower supervisory and technical occupations	0.098	0.832	7.08	6.65	7.52	1436
Semi‐routine occupations	0.092	0.765	24.48	23.7	25.28	5183
Missing	0.240	1.832	2.13	1.87	2.41	385
Equivalised income quintiles						
Lowest quintile (≤£14,918)	0.087	0.829	22.50	21.55	23.49	4440
Second lowest quintile (>£14,918 to ≤£23,084)	0.090	0.744	21.15	20.21	22.13	4231
Middle quintile (>£23,084 to ≤£31,967)	0.065	0.640	17.87	17	18.76	3758
Second highest quintile (>£31,967 to ≤£52 817)	0.109	0.916	13.52	12.76	14.32	2869
Highest quintile (>£52,817)	0.092	0.795	11.93	11.19	12.71	2279
Missing income	0.090	0.861	13.03	12.24	13.85	2514
Tenure						
Buying with mortgage/loan	0.078	0.669	38.78	37.65	39.93	7556
Own home outright	0.049	0.588	34.96	33.89	36.05	7852
Part rent/part mortgage	0.180	0.725	0.71	0.54	0.94	143
Rent (including rents paid by housing benefit)	0.161	1.157	24.66	23.62	25.72	4361
Living rent free	0.002	0.045	0.89	0.68	1.17	179
Number of cars normally available						
None	0.173	1.199	9.34	8.64	10.07	1660
One	0.091	0.837	38.52	37.4	39.66	8217
Two	0.071	0.621	41.47	40.34	42.6	8349
Three or more	0.074	0.781	10.68	9.93	11.47	1865
Weekly drinking category						
Non‐drinker	0.076	0.741	12.47	11.8	13.16	2524
Moderate (men, <22 units; women, <15 units)	0.093	0.833	67.56	66.64	68.48	13 468
Hazardous (men, 22–50 units; women, 15–35 units)	0.078	0.544	15.74	15.08	16.44	3275
Harmful (men, >50 units; women, >35 units)	0.092	1.054	4.22	3.87	4.61	822
Cigarette smoking status						
Never smoked cigarettes at all	0.083	0.817	51.69	50.75	52.64	10 135
Used to smoke cigarettes occasionally	0.111	0.808	5.87	5.47	6.3	1180
Used to smoke cigarettes regularly	0.068	0.625	28.28	27.48	29.1	5962
Current cigarette smoker	0.139	0.980	14.16	13.45	14.89	2812
Ever exposed to passive smoke in own or others home						
Never exposed	0.080	0.771	88.74	88.07	89.38	17 749
Exposed	0.155	0.952	11.26	10.62	11.93	2340
Ever had high blood pressure (also known as hypertension)						
Yes	0.058	0.577	24.38	23.65	25.13	5418
No	0.098	0.852	75.62	74.87	76.35	14 673
Ever had diabetes						
Yes	0.065	0.717	6.67	6.27	7.1	1420
No	0.090	0.799	93.33	92.9	93.73	18 671
Limiting long‐lasting illness						
Limiting long‐lasting illness	0.075	0.733	21.59	20.86	22.34	5016
Non‐limiting long‐lasting illness	0.064	0.586	18.58	17.91	19.26	3754
No limiting long‐lasting illness	0.100	0.868	59.83	58.92	60.74	11 321
Marital status recoded						
Married/civil partnership	0.070	0.756	79.42	78.39	80.4	16 553
Living as married	0.159	0.921	20.58	19.6	21.61	3538
Household type						
Small family: 2 adults of any age and 1 or 2 children	0.121	0.928	23.08	22.14	24.06	4684
Older smaller family: 1 or more adults of 65+ years and 1 or 2 children	0.023	0.344	22.81	21.93	23.71	5659
Large adult: 3+ adults, no children	0.063	0.669	15.40	14.48	16.37	2322
Small adult: 2 adults under 65 years and no children	0.100	0.768	27.36	26.31	28.43	5431
Large family: 2 adults of any age and 3+ children or 3+ adults and 1+ children	0.159	1.233	11.35	10.59	12.15	1995
Quintiles of Indices of Multiple Deprivation (IMD) score						
Least deprived quintile	0.050	0.498	23.00	22.08	23.96	4801
2nd	0.073	0.656	22.16	21.22	23.13	4786
3rd	0.069	0.743	21.96	21	22.96	4379
4th	0.119	0.918	18.40	17.49	19.35	3503
Most deprived quintile	0.162	1.175	14.47	13.66	15.33	2622
Government Office Region						
North East	0.166	1.429	4.26	4.16	4.37	1043
North West	0.090	0.615	11.97	11.71	12.24	1792
Yorkshire and the Humber	0.113	0.918	8.76	8.58	8.95	1187
East Midlands	0.068	0.520	8.40	8.24	8.57	1302
West Midlands	0.072	0.835	9.66	9.45	9.88	1232
East of England	0.063	0.492	10.73	10.51	10.95	1658
London	0.103	0.923	12.22	11.89	12.57	1309
South East	0.078	0.670	15.21	14.96	15.46	2187
South West	0.060	0.586	10.11	9.92	10.31	1452
Scotland	0.124	1.077	8.67	8.56	8.78	6929
Rurality of dwelling unit (urban/rural) – binary – recoded						
Urban	0.095	0.840	78.33	77.43	79.2	15 024
Town/fringe/village, hamlet and isolated dwellings	0.065	0.596	21.67	20.8	22.57	5067
Whether spent money on any gambling activity in last 12 months						
Yes, spent money on one or more gambling activities	0.095	0.797	60.77	59.82	61.72	12 450
Did not spend money on gambling activities in past year	0.078	0.789	39.23	38.28	40.18	7641
Total	0.088	0.794	100.00			20 091

*Note*: Base: individuals with Problem Gambling Severity Index (PGSI) = 0 who are in a cohabiting couple.

Table 2 shows the PGSI scores for the partners of the sample members (the individuals whose own PGSI scores were equal to zero). The partners of the sample members had a mean PGSI score of 0.088 (SD = 0.794).

**TABLE 2:** Partner Problem Gambling Severity Index (PGSI) scores of the sample members.

Partner PGSI scores	
Mean	0.088
Standard deviation	0.794
Unweighted base	20 091

*Note*: Base: individuals with PGSI = 0 who are in a cohabiting couple.

**FIGURE 1:**
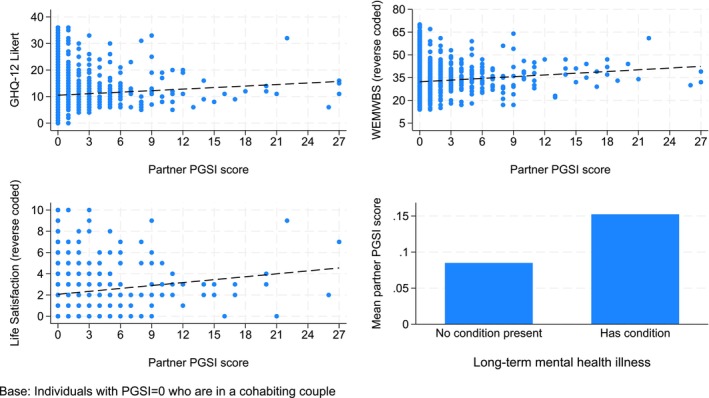
PGSI and four measures of an individual’s emotional wellbeing. GHQ‐12, 12‐item General Health Questionnaire; PGSI, Problem Gambling Severity Index; WEMWBS, Warwick Edinburgh Mental Health and Wellbeing Scale.

Table 3a shows the correlation between three emotional wellbeing indicators for the individuals and their partner’s PGSI scores. As their partner’s PGSI score increases, the individual’s scores on each measure worsen. This pattern can also be seen in the scatter plots in Figure 1. The plots use the reverse‐coded WEMWBS and life satisfaction scores, with increasing trend lines indicating a decline in wellbeing as the partner PGSI score increases.

**TABLE 3a:** Mean emotional wellbeing scores of the individual and correlation with partner PGSI.

	Mean wellbeing score of the individual	Standard deviation of individual's wellbeing score	Correlation of individual's wellbeing score with partner PGSI	*P*‐value of correlation	Unweighted base
WEMWBS score	51.98	8.21	−0.042	<0.001	15 381
GHQ‐12 score	10.65	4.73	0.036	<0.001	16 523
Overall satisfaction with life nowadays	7.75	1.79	−0.045	<0.001	13 491

*Note*: Base: individuals with PGSI = 0 who are in a cohabiting couple.

Abbreviations: GHQ‐12, 12‐item General Health Questionnaire; PGSI, Problem Gambling Severity Index; WEMWBS, Warwick Edinburgh Mental Health and Wellbeing Scale.

Table 3b shows individuals with and without a long‐term mental health condition and their partner’s mean PGSI score. Individuals with a long‐term mental health condition have partners with a higher mean PGSI score than individuals who have no long‐term mental health condition (see Figure 1).

**TABLE 3b:** Mean partner PGSI score for individuals with and without a long‐term mental health condition.

Long‐term mental health	Mean partner PGSI	Standard deviation of partner PGSI	Unweighted base
No condition present	0.085	0.781	16 067
Has condition	0.152	0.995	1015
Total	0.088	0.794	20 082

*Note*: Base: Individuals with PGSI = 0 who are in a cohabiting couple.

Abbreviations: PGSI, Problem Gambling Severity Index.

The unadjusted regression models show statistically significant associations between the full range of partner PGSI scores and each measure of emotional wellbeing: WEMWBS (*P* <0.001, coef. = 0.036; 95% CI = 0.017–0.055), GHQ‐12 Likert scale (*P* = 0.001, coef. = 0.039, 95% CI = 0.015–0.062) and life satisfaction (*P* = 0.002, coef. = 0.053, 95% CI = 0.020–0.086). In each instance, there is an association between individuals having poorer wellbeing outcomes and their partner having an elevated PGSI score. The adjusted regression models show that the association remains significant and in the same direction after controls were added: WEMWBS (*P* = 0.018, coef. = 0.022, 95% CI = 0.004–0.040), GHQ‐12 Likert scale (*P* = 0.048, coef. = 0.021, 95% CI = 0.000–0.043) and life satisfaction (*P* = 0.036, coef. = 0.036, 95% CI = 0.005–0.067).

As the analysis used standardised emotional wellbeing scores with a mean of zero and a standard deviation of one, the size of the coefficients from each model could be compared. The coefficients for life satisfaction were larger than the coefficients for WEMWBS and GHQ‐12, implying that the impact of partner PGSI was greatest on life satisfaction. Tables 4a, 4b, 4c, 4d show the output for the full models that include the control variables.

**TABLE 4a:** Unadjusted and adjusted regression output: Warwick Edinburgh Mental Health and Wellbeing Scale (WEMWBS).

Baseline	Categories	Coef.	95% CI	*Z*‐test	*P*‐value
**Unadjusted**					
Partner PGSI score		0.036	0.017, 0.055	3.743	<0.001
Constant		−0.032	−0.055, –0.009	−2.777	0.005
**Adjusted**					
Partner PGSI score		0.022	0.004, 0.040	2.367	0.018
Individual had not spent money on any gambling activity in last 12 months	Individual had spent money on any gambling activity in last 12 months	0.008	−0.033, 0.049	0.384	0.701
16–24 years	25–34 years	−0.099	−0.224, 0.027	−1.542	0.123
	35–44 years	0.007	−0.127, 0.140	0.101	0.920
	45–54 years	0.008	−0.127, 0.143	0.110	0.912
	55–64 years	−0.131	−0.272, 0.010	−1.821	0.069
	65–74 years	−0.220	−0.394, –0.045	−2.470	0.014
	75+ years	−0.095	−0.289, 0.099	−0.960	0.337
Male	Female	0.008	−0.028, 0.044	0.435	0.664
Other ethnic background	White	0.211	0.108, 0.315	3.995	<0.001
No religion	Christian – Catholic	−0.069	−0.126, –0.012	−2.363	0.018
	Christian – all other denominations	−0.051	−0.097, –0.005	−2.155	0.031
	Any other religion	0.040	−0.074, 0.155	0.690	0.490
In employment, self‐employment or government training	In full‐time education	0.003	−0.168, 0.174	0.038	0.970
	Retired	−0.139	−0.218, –0.060	−3.469	0.001
	ILO unemployed	0.123	−0.014, 0.260	1.754	0.079
	Other inactive	0.179	0.100, 0.258	4.439	<0.001
Degree (or equivalent) or higher	Higher education below degree	0.044	−0.019, 0.107	1.384	0.167
	A‐Level/Scottish Higher/or equivalent	0.106	0.045, 0.167	3.399	0.001
	GCSE/Scottish Standard Grade/or equivalent	0.183	0.121, 0.244	5.826	<0.001
	Other	0.117	−0.041, 0.276	1.452	0.146
	No qualifications	0.158	0.082, 0.233	4.103	<0.001
Missing occupation	Managerial and professional occupations	0.154	−0.012, 0.320	1.823	0.068
	Intermediate occupations	0.256	0.087, 0.426	2.963	0.003
	Small employers and own account workers	0.082	−0.091, 0.256	0.930	0.352
	Lower supervisory and technical occupations	0.208	0.030, 0.386	2.297	0.022
	Semi‐routine occupations	0.193	0.028, 0.359	2.294	0.022
Missing income	Lowest quintile (≤£14,918)	−0.051	−0.132, 0.030	−1.239	0.216
	Second lowest quintile (>£14,918 to ≤£23,084)	0.017	−0.064, 0.097	0.403	0.687
	Middle quintile (>£23,084 to ≤£31,967)	−0.035	−0.116, 0.046	−0.849	0.396
	Second highest quintile (>£31,967 to ≤£52 817)	0.056	−0.033, 0.146	1.233	0.217
	Highest quintile (>£52,817)	0.143	0.042, 0.243	2.777	0.006
Buying with mortgage/loan	Own it outright	−0.030	−0.077, 0.017	−1.249	0.212
	Part rent/part mortgage	0.209	−0.047, 0.466	1.600	0.110
	Rent (including rents paid by housing benefit)	−0.016	−0.079, 0.047	−0.489	0.625
	Living rent free	−0.149	−0.387, 0.090	−1.221	0.222
No cars available in household	One	−0.008	−0.100, 0.084	−0.178	0.859
	Two	−0.065	−0.160, 0.031	−1.330	0.184
	Three or more	−0.111	−0.228, 0.007	−1.851	0.064
Non‐drinker	Moderate (men, <22 units; women, <15 units)	−0.083	−0.155, –0.011	−2.262	0.024
	Hazardous (men, 22–50 units; women, 15–35 units)	−0.093	−0.176, –0.010	−2.197	0.028
	Harmful (men, >50 units; women, >35 units)	0.036	−0.084, 0.157	0.588	0.557
Never smoked cigarettes at all	Used to smoke cigarettes occasionally	0.056	−0.023, 0.136	1.386	0.166
	Used to smoke cigarettes regularly	0.042	−0.003, 0.087	1.846	0.065
	Current cigarette smoker	0.095	0.029, 0.161	2.801	0.005
Never exposed to tobacco smoke	Exposed	0.074	0.007, 0.141	2.172	0.030
Has high blood pressure (hypertension)	Does not have high blood pressure (hypertension)	−0.068	−0.115, –0.021	−2.834	0.005
Has diabetes	Does not have diabetes	−0.049	−0.131, 0.032	−1.188	0.235
Limiting long‐lasting illness	Non‐limiting long‐lasting illness	−0.532	−0.595, –0.469	−16.595	<0.001
	No limiting long‐lasting illness	−0.635	−0.691, –0.578	−22.005	<0.001
Married/civil partnership	Living as married	0.082	0.020, 0.143	2.599	0.009
Small family: 2 adults of any age and 1 or 2 children	Older small family: 1 or more adults aged 65+ years and 1 or 2 children	−0.083	−0.198, 0.033	−1.403	0.161
	Large adult: 3+ adults, no children	0.098	0.010, 0.187	2.179	0.029
	Small adult: 2 adults under 65 and no children	0.004	−0.059, 0.067	0.133	0.894
	Large family: 2 adults of any age and 3+ children or 3+ adults and 1+ children	−0.021	−0.103, 0.060	−0.512	0.609
Least deprived	2nd	0.030	−0.027, 0.087	1.038	0.299
	3rd	0.011	−0.048, 0.070	0.370	0.711
	4th	0.035	−0.030, 0.100	1.051	0.293
	Most deprived	0.030	−0.049, 0.108	0.742	0.458
Urban	Town/fringe/village, hamlet and isolated dwellings	−0.005	−0.056, 0.045	−0.209	0.835
North East	North West	−0.025	−0.133, 0.084	−0.449	0.653
	Yorkshire and the Humber	0.035	−0.081, 0.152	0.593	0.553
	East Midlands	0.004	−0.110, 0.118	0.068	0.946
	West Midlands	0.004	−0.112, 0.121	0.075	0.940
	East of England	0.041	−0.068, 0.150	0.733	0.464
	London	0.013	−0.111, 0.136	0.199	0.842
	South East	−0.040	−0.143, 0.063	−0.761	0.447
	South West	0.047	−0.062, 0.156	0.840	0.401
	Scotland	0.105	0.011, 0.199	2.179	0.029
General health very good/good	Fair	–	–	–	–
	Bad/very bad	–	–	–	–
Survey year (2012)	2015	0.100	0.048,0.152	3.774	<0.001
	2016	0.289	0.237,0.342	10.745	<0.001
	2017/8	0.169	0.093,0.244	4.363	<0.001
Constant		0.087	−0.210,0.385	0.575	0.566
Base (unweighted)		15 379			

**TABLE 4b:** Unadjusted and adjusted regression output: 12‐item General Health Questionnaire (GHQ‐12).

Baseline	Categories	Coef.	95% CI	*Z*‐test	*P*‐value
**Unadjusted**					
Partner PGSI score		0.039	0.015, 0.062	3.262	0.001
Constant		0.023	0.002, 0.044	2.137	0.033
**Adjusted**					
Partner PGSI score		0.021	0.000, 0.043	1.974	0.048
Individual had not spent money on any gambling activity in last 12 months	Individual had spent money on any gambling activity in last 12 months	−0.006	−0.046, 0.034	−0.296	0.767
16–24 years	25–34 years	0.036	−0.095, 0.168	0.542	0.588
	35–44 years	0.169	0.032, 0.306	2.424	0.015
	45–54 years	0.122	−0.018, 0.262	1.704	0.088
	55–64 years	0.036	−0.109, 0.180	0.485	0.628
	65–74 years	−0.039	−0.215, 0.137	−0.434	0.664
	75+ years	−0.036	−0.226, 0.155	−0.365	0.715
Male	Female	0.116	0.079, 0.154	6.081	<0.001
Other ethnic background	White	0.203	0.101, 0.305	3.902	<0.001
No religion	Christian – Catholic	−0.037	−0.092, 0.018	−1.313	0.189
	Christian – all other denominations	−0.007	−0.050, 0.037	−0.304	0.761
	Any other religion	0.102	−0.023, 0.227	1.596	0.111
In employment, self‐employment or government training	In full‐time education	0.210	0.020, 0.400	2.166	0.030
	Retired	−0.121	−0.193, –0.049	−3.286	0.001
	ILO unemployed	0.342	0.155, 0.530	3.587	<0.001
	Other inactive	0.351	0.266, 0.435	8.096	<0.001
Degree (or equivalent) or higher	Higher education below degree	−0.018	−0.078, 0.043	−0.575	0.566
	A‐Level/Scottish Higher/or equivalent	−0.046	−0.108, 0.017	−1.434	0.152
	GCSE/Scottish Standard Grade/or equivalent	−0.021	−0.080, 0.039	−0.681	0.496
	Other	−0.123	−0.283, 0.037	−1.511	0.131
	No qualifications	0.009	−0.068, 0.085	0.221	0.825
Missing occupation	Managerial and professional occupations	0.165	−0.016, 0.347	1.784	0.075
	Intermediate occupations	0.198	0.012, 0.384	2.083	0.037
	Small employers and own account workers	0.159	−0.031, 0.350	1.641	0.101
	Lower supervisory and technical occupations	0.117	−0.074, 0.307	1.198	0.231
	Semi‐routine occupations	0.117	−0.065, 0.300	1.257	0.209
Missing income	Lowest quintile (≤£14,918)	0.034	−0.035, 0.104	0.965	0.334
	Second lowest quintile (>£14,918 to ≤£23,084)	0.046	−0.024, 0.115	1.291	0.197
	Middle quintile (>£23,084 to ≤£31,967)	0.040	−0.030, 0.110	1.110	0.267
	Second highest quintile (>£31,967 to ≤£52 817)	0.048	−0.028, 0.125	1.238	0.216
	Highest quintile (>£52,817)	0.188	0.099, 0.277	4.128	<0.001
Buying with mortgage/loan	Own it outright	−0.030	−0.075, 0.014	−1.337	0.181
	Part rent/part mortgage	0.100	−0.138, 0.337	0.824	0.410
	Rent (including rents paid by housing benefit)	−0.042	−0.104, 0.020	−1.340	0.180
	Living rent free	−0.020	−0.234, 0.195	−0.179	0.858
No cars available in household	One	0.019	−0.067, 0.105	0.430	0.667
	Two	−0.020	−0.109, 0.069	−0.450	0.653
	Three or more	−0.010	−0.123, 0.103	−0.180	0.857
Non‐drinker	Moderate (men, <22 units; women, <15 units)	−0.081	−0.154, –0.009	−2.194	0.028
	Hazardous (men, 22–50 units; women, 15–35 units)	−0.091	−0.173, –0.008	−2.141	0.032
	Harmful (men, >50 units; women, >35 units)	−0.008	−0.127, 0.110	−0.139	0.889
Never smoked cigarettes at all	Used to smoke cigarettes occasionally	0.110	0.027, 0.194	2.604	0.009
	Used to smoke cigarettes regularly	0.008	−0.034, 0.051	0.375	0.708
	Current cigarette smoker	0.020	−0.044, 0.085	0.616	0.538
Never exposed to tobacco smoke	Exposed	0.090	0.023, 0.158	2.611	0.009
Has high blood pressure (hypertension)	Does not have high blood pressure (hypertension)	−0.089	−0.137, –0.041	−3.648	<0.001
Has diabetes	Does not have diabetes	−0.059	−0.148, 0.031	−1.282	0.200
Limiting long‐lasting illness	Non‐limiting long‐lasting illness	−0.653	−0.716, –0.590	−20.292	<0.001
	No limiting long‐lasting illness	−0.755	−0.812, –0.698	−25.849	<0.001
Married/civil partnership	Living as married	0.061	0.002, 0.120	2.016	0.044
Small family: 2 adults of any age and 1 or 2 children	Older small family: 1 or more adults aged 65+ years and 1 or 2 children	−0.049	−0.156, 0.059	−0.890	0.373
	Large adult: 3+ adults, no children	0.052	−0.028, 0.131	1.280	0.201
	Small adult: 2 adults under 65 and no children	0.048	−0.013, 0.110	1.540	0.124
	Large family: 2 adults of any age and 3+ children or 3+ adults and 1+ children	0.055	−0.021, 0.131	1.419	0.156
Least deprived	2nd	0.040	−0.011, 0.092	1.542	0.123
	3rd	0.029	−0.027, 0.085	1.009	0.313
	4th	0.043	−0.019, 0.105	1.353	0.176
	Most deprived	−0.001	−0.078, 0.075	−0.036	0.971
Urban	Town/fringe/village, hamlet and isolated dwellings	−0.016	−0.062, 0.030	−0.675	0.500
North East	North West	−0.063	−0.164, 0.038	−1.217	0.224
	Yorkshire and the Humber	0.018	−0.090, 0.127	0.335	0.738
	East Midlands	0.027	−0.079, 0.133	0.499	0.617
	West Midlands	0.045	−0.062, 0.152	0.821	0.412
	East of England	−0.006	−0.108, 0.096	−0.110	0.912
	London	−0.049	−0.164, 0.065	−0.847	0.397
	South East	0.004	−0.094, 0.102	0.081	0.936
	South West	−0.007	−0.107, 0.092	−0.146	0.884
	Scotland	−0.116	−0.205, –0.027	−2.543	0.011
General health very good/good	Fair	–	–	–	–
	Bad/Very bad	–	–	–	–
Survey year (2012)	2015	0.032	−0.037, 0.100	0.905	0.365
	2016	0.07	0.021, 0.118	2.803	0.005
	2017/8	0.005	−0.042, 0.053	0.222	0.824
Constant		0.228	−0.073, 0.528	1.487	0.137
Base (unweighted)		16 521			

**TABLE 4c:** Unadjusted and adjusted regression output: life satisfaction.

Baseline	Categories	Coef.	95% CI	*Z*‐test	*P*‐value
**Unadjusted**					
Partner PGSI score		0.053	0.020, 0.086	3.151	0.002
Constant		0.097	0.070, 0.124	7.053	<0.001
**Adjusted**					
Partner PGSI score		0.036	0.005, 0.067	2.291	0.022
Individual had not spent money on any gambling activity in last 12 months	Individual had spent money on any gambling activity in last 12 months	0.019	−0.028, 0.067	0.802	0.423
16–24 years	25–34 years	0.047	−0.113, 0.207	0.577	0.564
	35–44 years	0.145	−0.022, 0.313	1.699	0.089
	45–54 years	0.152	−0.016, 0.321	1.771	0.077
	55–64 years	0.039	−0.133, 0.212	0.445	0.656
	65–74 years	−0.068	−0.278, 0.142	−0.638	0.523
	75+ years	−0.014	−0.244, 0.217	−0.115	0.908
Male	Female	−0.036	−0.078, 0.006	−1.661	0.097
Other ethnic background	White	0.102	−0.013, 0.216	1.744	0.081
No religion	Christian – Catholic	−0.089	−0.156, –0.022	−2.592	0.010
	Christian all other denominations	−0.096	−0.149, –0.042	−3.527	<0.001
	Any other religion	0.080	−0.060, 0.220	1.123	0.261
In employment, self‐employment or government training	In full‐time education	0.034	−0.156, 0.223	0.346	0.729
	Retired	−0.108	−0.195, –0.021	−2.440	0.015
	ILO unemployed	0.514	0.313, 0.715	5.012	<0.001
	Other inactive	0.419	0.310, 0.527	7.586	<0.001
Degree (or equivalent) or higher	Higher education below degree	−0.013	−0.086, 0.059	−0.362	0.717
	A‐Level/Scottish Higher/or equivalent	−0.027	−0.098, 0.044	−0.743	0.458
	GCSE/Scottish Standard Grade/or equivalent	−0.050	−0.119, 0.019	−1.420	0.156
	Other	−0.053	−0.267, 0.161	−0.486	0.627
	No qualifications	−0.035	−0.129, 0.058	−0.744	0.457
Missing occupation	Managerial and professional occupations	0.251	0.034, 0.469	2.269	0.023
	Intermediate occupations	0.283	0.062, 0.504	2.510	0.012
	Small employers and own account workers	0.293	0.067, 0.518	2.542	0.011
	Lower supervisory and technical occupations	0.177	−0.051, 0.406	1.521	0.128
	Semi‐routine occupations	0.291	0.075, 0.508	2.634	0.008
Missing income	Lowest quintile (≤£14,918)	−0.127	−0.214, –0.040	−2.851	0.004
	Second lowest quintile (>£14,918 to ≤£23,084)	−0.056	−0.147, 0.034	−1.218	0.223
	Middle quintile (>£23,084 to ≤£31,967)	−0.118	−0.208, –0.027	−2.556	0.011
	Second highest quintile (>£31,967 to ≤£52 817)	−0.030	−0.128, 0.068	−0.600	0.548
	Highest quintile (>£52,817)	0.001	−0.113, 0.116	0.021	0.983
Buying with mortgage/loan	Own it outright	−0.001	−0.053, 0.051	−0.034	0.973
	Part rent/part mortgage	0.132	−0.166, 0.431	0.869	0.385
	Rent (including rent paid by housing benefit)	0.054	−0.024, 0.133	1.358	0.175
	Living rent free	0.013	−0.214, 0.241	0.116	0.908
No cars available in household	One	−0.045	−0.160, 0.069	−0.776	0.438
	Two	−0.130	−0.247, –0.014	−2.194	0.028
	Three or more	−0.126	−0.266, 0.015	−1.753	0.080
Non‐drinker	Moderate (men, <22 units; women, <15 units)	−0.054	−0.140, 0.033	−1.218	0.223
	Hazardous (men, 22–50 units; women, 15–35 units)	−0.067	−0.166, 0.032	−1.335	0.182
	Harmful (men, >50 units; women, >35 units)	0.029	−0.116, 0.175	0.395	0.693
Never smoked cigarettes at all	Used to smoke cigarettes occasionally	0.054	−0.043, 0.152	1.089	0.276
	Used to smoke cigarettes regularly	0.025	−0.024, 0.075	1.005	0.315
	Current cigarette smoker	0.074	−0.008, 0.157	1.760	0.078
Never exposed to tobacco smoke	Exposed	0.097	0.014, 0.180	2.283	0.022
Has high blood pressure (hypertension)	Does not have high blood pressure (hypertension)	−0.059	−0.115, –0.003	−2.078	0.038
Has diabetes	Does not have diabetes	−0.057	−0.151, 0.037	−1.188	0.235
Limiting long‐lasting illness	Non‐limiting long‐lasting illness	−0.658	−0.728, –0.587	−18.250	<0.001
	No limiting long‐lasting illness	−0.725	−0.790, –0.659	−21.680	<0.001
Married/civil partnership	Living as married	0.061	−0.011, 0.132	1.659	0.097
Small family: 2 adults of any age and 1 or 2 children	Older small family: 1 or more adults aged 65+ years and 1 or 2 children	−0.018	−0.147, 0.112	−0.269	0.788
	Large adult: 3+ adults, no children	0.117	0.010, 0.223	2.140	0.032
	Small adult: 2 adults under 65 and no children	0.045	−0.030, 0.120	1.170	0.242
	Large family: 2 adults of any age and 3+ children or 3+ adults and 1+ children	0.088	−0.012, 0.189	1.722	0.085
Least deprived	2nd	0.029	−0.037, 0.095	0.859	0.391
	3rd	0.010	−0.059, 0.079	0.275	0.783
	4th	0.059	−0.017, 0.135	1.526	0.127
	Most deprived	0.044	−0.050, 0.137	0.916	0.360
Urban	Town/fringe/village, hamlet and isolated dwellings	−0.046	−0.105, 0.013	−1.534	0.125
North East	North West	−0.029	−0.149, 0.092	−0.469	0.639
	Yorkshire and the Humber	0.112	−0.028, 0.253	1.570	0.117
	East Midlands	0.061	−0.065, 0.187	0.945	0.345
	West Midlands	0.070	−0.061, 0.200	1.049	0.294
	East of England	0.048	−0.080, 0.176	0.732	0.464
	London	0.001	−0.142, 0.144	0.014	0.989
	South East	0.046	−0.072, 0.164	0.762	0.446
	South West	0.046	−0.077, 0.170	0.735	0.462
	Scotland	−0.237	−0.342, –0.131	−4.403	<0.001
General health very good/good	Fair	–	–	–	–
	Bad/very bad	–	–	–	–
Survey year (2012)	2015	−0.036	−0.116, 0.045	−0.873	0.383
	2016	0.032	−0.044, 0.108	0.832	0.405
	2017/8	−0.121	−0.199, –0.044	−3.058	0.002
Constant		0.506	0.123, 0.889	2.591	0.010
Base (unweighted)		13 489			

**TABLE 4d:** Unadjusted and adjusted regression output: long‐term mental health condition.

Baseline	Categories	Odds ratio	95% CI	*Z*‐test	*P*‐value
**Unadjusted**					
Partner PGSI score		1.071	1.021,1.122	2.841	0.004
Constant		0.054	0.049,0.058	−70.814	<0.001
**Adjusted**					
Partner PGSI score		1.023	0.965,1.086	0.769	0.442
Individual had not spent money on any gambling activity in last 12 months	Individual had spent money on any gambling activity in last 12 months	0.796	0.671,0.944	−2.618	0.009
16–24 years	25–34 years	0.523	0.341,0.802	−2.975	0.003
	35–44 years	0.819	0.526,1.275	−0.886	0.376
	45–54 years	0.539	0.344,0.846	−2.691	0.007
	55–64 years	0.377	0.232,0.614	−3.925	<0.001
	65–74 years	0.490	0.233,1.030	−1.883	0.060
	75+ years	0.288	0.125,0.665	−2.918	0.004
Male	Female	1.593	1.339,1.895	5.265	<0.001
Other ethnic background	White	3.665	2.224,6.039	5.098	<0.001
No religion	Christian ‐ Catholic	0.608	0.470,0.788	−3.766	<0.001
	Christian all other denominations	0.784	0.645,0.953	−2.439	0.015
In employment, self‐employment or government training	Any other religion	1.051	0.682,1.619	0.225	0.822
	In full‐time education	0.510	0.218,1.198	−1.545	0.122
	Retired	0.984	0.666,1.455	−0.080	0.936
	ILO unemployed	1.561	0.972,2.508	1.843	0.065
	Other inactive	2.170	1.730,2.722	6.701	<0.001
Degree (or equivalent) or higher	Higher education below degree	0.906	0.665,1.234	−0.625	0.532
	A‐level/Scottish highers/or equivalent	1.059	0.806,1.392	0.413	0.680
	GCSE/Scottish Standard Grades/or equivalent	1.034	0.792,1.349	0.244	0.807
	Other	0.590	0.220,1.585	−1.046	0.296
	No qualifications	0.822	0.586,1.151	−1.142	0.253
Missing occupation	Managerial and professional occupations	0.684	0.409,1.143	−1.450	0.147
	Intermediate occupations	0.836	0.503,1.390	−0.690	0.490
	Small employers and own account workers	0.785	0.453,1.357	−0.868	0.386
	Lower supervisory and technical occupations	0.565	0.317,1.007	−1.938	0.053
	Semi‐routine occupations	0.758	0.460,1.247	−1.092	0.275
Missing income	Lowest quintile (≤£14,918)	1.188	0.821,1.718	0.915	0.360
	Second lowest quintile (>£14,918 to ≤£23,084)	1.394	0.991,1.962	1.906	0.057
	Middle quintile (>£23,084 to ≤£31,967)	1.611	1.157,2.243	2.824	0.005
	Second highest quintile (>£31,967 to ≤£52 817)	1.750	1.248,2.452	3.249	0.001
	Highest quintile (>£52,817)	1.754	1.242,2.478	3.190	0.001
Buying with mortgage/loan	Own it outright	0.973	0.777,1.218	−0.237	0.813
	Part rent/part mortgage	0.999	0.457,2.182	−0.004	0.997
	Rent (including rents paid by housing benefit)	1.129	0.892,1.430	1.010	0.313
	Living here rent free	2.355	0.988,5.613	1.932	0.053
No cars available in household	One	0.928	0.703,1.225	−0.528	0.598
	Two	0.861	0.631,1.174	−0.947	0.344
	Three or more	0.941	0.606,1.462	−0.270	0.787
Non‐drinker	Moderate (men, <22 units; women, <15 units)	0.762	0.597,0.974	−2.173	0.030
	Hazardous (men, 22–50 units; women, 15–35 units)	0.704	0.514,0.966	−2.176	0.030
	Harmful (men, >50 units; women, >35 units)	1.270	0.855,1.886	1.186	0.236
Never smoked cigarettes at all	Used to smoke cigarettes occasionally	1.337	0.951,1.879	1.673	0.094
	Used to smoke cigarettes regularly	1.440	1.182,1.756	3.612	<0.001
	Current cigarette smoker	1.522	1.180,1.963	3.230	0.001
Never exposed to tobacco smoke	Exposed	0.947	0.728,1.231	−0.408	0.683
Has high blood pressure (hypertension)	Does not have high blood pressure (hypertension)	0.912	0.755,1.102	−0.954	0.340
Has diabetes	Does not have diabetes	1.378	1.001,1.897	1.967	0.049
Limiting long‐lasting illness	Non‐limiting long‐lasting illness				
	No limiting long‐lasting illness				
Married/civil partnership	Living as married	0.973	0.777,1.218	−0.239	0.811
Small family: 2 adults of any age and 1 or 2 children	Older small family: 1 or more adults aged 65+ years and 1 or 2 children	0.635	0.364,1.107	−1.602	0.109
	Large adult: 3+ adults, no children	1.196	0.880,1.627	1.144	0.253
	Small adult: 2 adults under 65 and no children	1.342	1.035,1.742	2.216	0.027
	Large family: 2 adults of any age and 3+ children or 3+ adults and 1+ children	1.002	0.732,1.371	0.013	0.990
Least deprived	2nd	1.252	0.959,1.634	1.655	0.098
	3rd	1.002	0.756,1.328	0.013	0.990
	4th	1.142	0.856,1.524	0.902	0.367
	Most deprived	1.046	0.763,1.435	0.281	0.779
Urban	Town/Fringe/Village, hamlet and isolated dwellings	1.061	0.855,1.316	0.535	0.592
North East	North West	1.236	0.829,1.843	1.041	0.298
	Yorkshire and the Humber	1.306	0.872,1.955	1.296	0.195
	East Midlands	1.263	0.835,1.911	1.105	0.269
	West Midlands	1.206	0.799,1.821	0.893	0.372
	East of England	1.514	1.030,2.226	2.110	0.035
	London	0.825	0.491,1.386	−0.725	0.468
	South East	1.241	0.845,1.822	1.100	0.271
	South West	1.433	0.951,2.161	1.721	0.085
	Scotland	1.162	0.830,1.628	0.875	0.382
General health very good/good	Fair	1.43	1.110,1.842	2.772	0.006
	Bad/Very bad	1.73	1.350,2.216	4.335	<0.001
Survey year (2012)	2015	1.905	1.489,2.436	5.133	<0.001
	2016	3.957	3.253,4.814	13.755	<0.001
	2017/8	5.894	4.532,7.666	13.23	<0.001
Constant		0.006	0.002,0.018	−9.663	<0.001
Base (unweighted)		20 082			

The mean partner PGSI for people with a long‐term mental health condition was 0.152 (SD = 0.995), higher than the respective mean for people without a mental health condition (mean = 0.085, SD = 0.781). The unadjusted regression models indicated that this difference was statistically significant (*P* = 0.004, OR = 1.071, 95% CI = 1.021–1.122); however, the association in the adjusted regression models is not statistically significant (*P* = 0.442, OR = 1.023, 95% CI = 0.965–1.086).

## DISCUSSION

This analysis looked at the impact of a spouse or cohabiting partner’s PGSI score on a set of four emotional and mental health measures for individuals whose own PGSI score was equal to zero, and who were therefore considered to be at low risk of harm from their own gambling.

It showed that there was an association between increases in their partner’s PGSI score and declining levels of their own emotional wellbeing, measured by WEMWBS, GHQ‐12 and life satisfaction score. This association remained after controls were included in the model, suggesting it is not explained by underlying differences in the socio‐demographics of different sets of spouses. This finding reflects wider evidence of the impact of a partner’s gambling on the emotional wellbeing of others [28].

This analysis adds substantially to the existing evidence base, demonstrating an association between detriments to the mental health and wellbeing of individuals and any elevation of PGSI score among their partners. The models show that any increase in partner PGSI score impacts on emotional wellbeing. Previous research has focused on the impact of disordered gambling on the wellbeing of close contacts. More recent studies have looked at the fuller range of risk and found evidence of a link between a close contact’s lower risk gambling and others experiencing financial harms, but not health, wellbeing or social harms. This analysis shows there is an association between an individual’s own emotional health and their partner’s gambling for those of any risk designation. Whilst there is a growing literature looking at PGSI as a spectrum of harms for the individual, this study adds to the sparser body of evidence for associated harms to close contacts.

Whilst for WEMWBS, GHQ‐12 and life satisfaction the association remained significant when accounting for a range of socio‐demographic, economic and lifestyle factors, there is no clear evidence of an association between a partner’s PGSI score and the presence of a long‐term mental health condition once the controls were included in the model. In addition to these controls accounting for this association, it should be noted that this dichotomous question, unlike the other measures, does not measure severity and can encompass a range of conditions, which may influence the results. Additionally, the question covers a 12‐month period, unlike the emotional wellbeing measures, which are answered in respect to last 2 weeks (WEMWBS) or the last 4 weeks (GHQ‐12). Thus, our analyses appear to identify associations between immediate emotional impact and partner PGSI scores but not the impact on long‐term mental health. This, in turn, may be because most of the sample had lower PGSI scores, and a crisis point might need to be reached before the gambling behaviour makes an impact on longer term mental health.

There are some limitations to this study. The data are cross‐sectional with attendant issues for causal inference; it may be that having a spouse or partner with poor emotional wellbeing increases the likelihood of harmful gambling behaviour. The analysis uses secondary data, which limits the pool of control variables available. There may be other factors that would explain the association that are not included in the data set and remain as unmeasured confounding factors, such as the quality of the partner relationship, the extent of additional social structures or the number of years impacted by gambling harms. The analysis focuses on partners, driven by a desire to focus on a specific relationship and sample size. Partners are more likely to be impacted and these findings cannot be extrapolated to other relationships. Nevertheless, partners are important for this very reason; they are more likely to have shared finances and shared dependents that leave them vulnerable to harms, and it is important that this risk is understood. Despite combining years of survey data and focusing on a more common relationship type, the sample sizes available for the analysis are still relatively small: around 3% of the individuals had partners with PGSI scores greater than zero. Finally, some of the outcomes were missing in some survey years, reducing the sample size for these measures. There is a small risk of bias, mitigated by the weighting design and by including the survey year as a covariate.

## CONCLUSION

To date, gambling policy has tended to focus on those experiencing gambling disorder and the attendant societal impacts from this group. Our analysis demonstrates that decrements to an individual’s emotional wellbeing are strongly associated with the presence of a spouse or cohabiting partner with increased PGSI scores, with emotional wellbeing declining as their partner’s PGSI score increases. Consideration of the wider impacts of gambling at sub‐clinical levels is needed.

## AUTHOR CONTRIBUTIONS

**Sarah Tipping:** Conceptualization (equal); data curation (lead); formal analysis (lead); investigation (lead); methodology (lead); project administration (lead); software (lead); validation (equal); writing—original draft (lead); writing—review and editing (equal). **Heather Wardle:** Conceptualization (equal); funding acquisition (lead); methodology (supporting); project administration (supporting); supervision (lead); validation (equal); writing—original draft (supporting); writing—review and editing (equal). **Robert Pryce:** Methodology (supporting); supervision (supporting); validation (equal); writing—review and editing (supporting).

## DECLARATION OF INTERESTS

In the last 3 years, H.W. discloses grant funding for gambling‐related research by the Economic and Social Research Council; National Institute for Health Research; Wellcome Trust; the Gambling Commission (including their regulatory settlement fund); the Office of Health Disparities and Improvements; Public Health England; Greater London Authority; Greater Manchester Combined Authority; Blackburn with Darwen Local Authority; and the Department of Digital Culture Media and Sport. H.W. declares consulting fees from the Institute of Public Health, Ireland, and the National Institute for Economic and Social Research. H.W. also declares payment for the delivery of seminars from McGill University, the University of Birmingham, John Hopkins University and from the British Broadcasting Corporation. H.W. has been paid as an expert witness by Lambeth and Middlesborough Borough Councils. H.W. also declares travel costs paid by Gambling Regulators European Forum, the Turkish Green Crescent Society, Alberta Gambling Research Institute, the REITOX Academy (administered through the Austrian National Public Health Institute) and the University of Helsinki. H.W. served as Deputy Chair of the Advisory Board for Safer Gambling between 2015 and 2020, remunerated by the Gambling Commission, is a Member of the World Health Organization (WHO) panel on gambling (ongoing) and has provided unpaid advice on research to GamCare for their Safer Gambling Standard (until mid‐2021). H.W. runs a research consultancy for public and third‐sector bodies only. She has not, and does not, provide consultancy services to gambling industry actors. In researching the gambling industry and their practices, H.W. declares occasional attendance at events where gambling industry actors are present (including industry‐sponsored conferences).

In the last 3 years, S.T. declares funding for gambling projects from the National Institute of Health Research and the Gambling Commission.

As part of their work on the Gambling Survey for Great Britain (GSGB), H.W. and S.T. are required by the Gambling Commission (the funder) to participate in events disseminating research findings to their stakeholders, which includes the industry. Their attendance at events where industry is present is independently funded and does not involve collaborations or partnerships with industry.

P.R. has no declarations.

## Supporting information


**Appendix S1:** Combining data from the Health Survey for England (HSE) and Scottish Health Survey (SHeS): A technical note.

**Appendix S2:** Treatment of missing values.

**Appendix S3:** Supplementary results from Poisson analysis of GHQ‐12
**Table S3.1:** Unadjusted and adjusted regression output: GHQ‐12.

**Appendix S4:** E‐values for Partner PGSI.
**Table S4.1:** E‐values for partner PGSI (continuous outcomes).
**Table S4.2:** E‐values for partner PGSI (binary outcome).

**Appendix S5:** STROBE Statement—Checklist of items that should be included in reports of cohort studies.

## Data Availability

Data derived from public domain resources.The data that support the findings of this study are available under Special Licence from the UK Data Service. These data are in the public domain.The analysis used eight datasets in total. The following years from the Health Survey for England (HSE) and Scottish Health Survey (SHeS) were used:HSE 2018 https://doi.org/10.5255/UKDA-SN-8961-1HSE 2016 https://doi.org/10.5255/UKDA-SN-9084-1HSE 2015 https://doi.org/10.5255/UKDA-SN-8372-2HSE 2012 https://doi.org/10.5255/UKDA-SN-7480-2SHeS 2017 https://doi.org/10.5255/UKDA-SN-8398-1SHeS 2016 https://doi.org/10.5255/UKDA-SN-8290-1SHeS 2015 https://doi.org/10.5255/UKDA-SN-8100-1SHeS 2012 https://doi.org/10.5255/UKDA-SN-7417-4.

## References

[1] Langham E , Thorne H , Browne M , Donaldson P , Rose J , Rockloff M . Understanding gambling related harm: A proposed definition, conceptual framework, and taxonomy of harms. BMC Public Health. 2016;16(1):80. 10.1186/s12889-016-2747-0 26818137 PMC4728872

[2] Goodwin BC , Browne M , Rockloff M , Rose J . A typical problem gambler affects six others. Int Gambl Stud. 2017;17(2):276–289. 10.1080/14459795.2017.1331252

[3] Wenzel HG , Øren A , Bakken IJ . Gambling problems in the family – A stratified probability sample study of prevalence and reported consequences. BMC Public Health. 2008;8:412. 10.1186/1471-2458-8-412 19087339 PMC2625355

[4] Salonen AH , Alho H , Castrén S . The extent and type of gambling harms for concerned significant others: A cross‐sectional population study in Finland. Scand J Public Health. 2016;44(8):799–804. 10.1177/1403494816673529 28929933

[5] Li E , Browne M , Rawat V , Langham E , Rockloff M . Breaking bad: Comparing gambling harms among gamblers and affected others. J Gambl Stud. 2017;33(1):223–248. 10.1007/s10899-016-9632-8 27443306

[6] Wardle H , Ridout K , Tipping S , Wilson H , Maxineanu I , Hill S . Gambling Survey for Great Britain ‐ Annual report (2023): Official statistics. 2024.

[7] Dowling NA , Hawker CO , Merkouris SS , Rodda SN , Hodgins DC . Addressing gambling harm to affected others: A scoping review Melbourne, Australia: Victorian Responsible Gambling Foundation; 2021.

[8] Riley BJ , Harvey P , Crisp BR , Battersby M , Lawn S . Gambling‐related harm as reported by concerned significant others: A systematic review and meta‐synthesis of empirical studies. J Fam Stud. 2018;27(1):112–130. 10.1080/13229400.2018.1513856

[9] Tulloch C , Browne M , Hing N , Rockloff M , Hilbrecht M . How gambling harms the wellbeing of family and others: A review. Int Gambl Stud. 2022;22(3):522–540. 10.1080/14459795.2021.2002384

[10] Collard S , Davies S , Cross K . The family dynamics of gambling harm Personal Finance Research Centre, University of Bristol; 2023.

[11] Jeffrey L , Browne M , Rawat V , Langham E , Li E , Rockloff M . Til debt do us part: comparing gambling harms between gamblers and their spouses. Journal of Gambling Studies. 2019;35(3):1015–1034. 10.1007/s10899-019-09826-3 30701378

[12] Svensson J , Romild U , Shepherdson E . The concerned significant others of people with gambling problems in a national representative sample in Sweden – a 1 year follow‐up study. BMC Public Health. 2013;13(1):1087. 10.1186/1471-2458-13-1087 24261955 PMC3870974

[13] Ferland F , Blanchette‐Martin N , Côté M , Tremblay J , Kairouz S , Nadeau L , et al. Do the consequences experienced by the people in the life of a problem gambler differ based on the nature of their relationship with the gambler? J Gambl Stud. 2021;38(3):1075–1092. 10.1007/s10899-021-10058-7 34286413

[14] Lind K , Castrén S , Hagfors H , Salonen AH . Harm as reported by affected others: A population‐based cross‐sectional Finnish gambling 2019 study. Addict Behav. 2022;107263. 10.1016/j.addbeh.2022.107263 PMID: see also Castrén, S., Lind, K., Hagfors, H., & Salonen, A. H. (2021). Gambling‐related harms for affected others: A Finnish population‐based survey. International Journal of Environmental Research and Public Health, 18, 9564. 35134630

[15] Hing N , Russell AMT , Browne M , Rockloff M , Tulloch C , Rawat V , et al. Gambling‐related harms to concerned significant others: A national Australian prevalence study. J Behav Addict. 2022;11(2):361–372. 10.1556/2006.2022.00045 PMID: 35895474. 35895474 PMC9295213

[16] Tulloch C , Browne M , Hing N , Rockloff M , Hilbrecht M . How gambling harms others: The influence of relationship‐type and closeness on harm, health, and wellbeing. J Behav Addict. 2023;12(3):697–710. 10.1556/2006.2023.00036 PMID: 37450370. 37450370 PMC10562824

[17] Dowling NA , Rodda SN , Lubman DI , Jackson AC . The impacts of problem gambling on concerned significant others accessing web‐based counselling. Addictive Behaviors. 2014;39(8):1253–1257. 10.1016/j.addbeh.2014.04.011 24813552

[18] Browne M , Rockloff MJ . The dangers of conflating gambling‐related harm with disordered gambling. J. Behav. Addict. 2017;6(3):317–320. 10.1556/2006.6.2017.059 28889755 PMC5700733

[19] Delfabbro P , King D . Prevention paradox logic and problem gambling: Does low‐risk gambling impose a greater burden of harm than high‐risk gambling? J Behav Addict. 2017;6(2):163–167. 10.1556/2006.6.2017.022 28425779 PMC5520119

[20] Browne M , Volberg R , Rockloff M , Salonen AH . The prevention paradox applies to some but not all gambling harms: Results from a Finnish population‐representative survey. J Behav Addict. 2020;9(2):371–382. Published online 2020 Jul 7. 10.1556/2006.2020.00018 32644932 PMC8939417

[21] Tulloch C , Hing N , Browne M , Rockloff M . How gambling problems relate to health and wellbeing in Australian households: Evidence from the Household Income and Labour Dynamics of Australia Survey. Addict Behav. 2023;137:107 538. 10.1016/j.addbeh.2022.107538 36368277

[22] Newall P , Rawat V , Hing N , Browne M , Tulloch C , Russell AMT , et al. Gambling related harm to affected others: Lived experience differs by relationship type, gambling severity, life circumstances, and relationship factors. Int J Ment Health Addict. 2024. 10.1007/s11469-024-01417-7

[23] https://digital.nhs.uk/data-and-information/publications/statistical/health-survey-for-england

[24] https://www.gov.scot/collections/scottish-health-survey

[25] Rodrigues LS , da Silva LM , Barbosa ES , de Souza RM , Vianna ALS , da Silva JM , et al. Analysis of HIV‐1 protease inhibitor resistance mutations in protease and reverse transcriptase genes in a cohort of patients failing antiretroviral therapy in Brazil. HIV Med. 2004;5(6):398–403. 10.1186/1477-7525-5-63

[26] Goldberg DP. General Health Questionnaire‐12 (GHQ‐12) APA PsycTests. 1972; 10.1037/t00297-000

[27] Ferris J , Wynne H . The Canadian Problem Gambling Index: Final report Ottawa, ON: Canadian Centre on Substance Abuse; 2001.

[28] Public Health England . Gambling‐related harms: evidence review London: Public Health England; 2021.

