# Supplementary material for: Exploring the impact on emotional wellbeing of having a spouse or cohabiting partner with elevated Problem Gambling Severity Index (PGSI) scores: Secondary analysis of cross‐sectional health survey data
Source: Addiction. 2025 Sep 3;120(12):2500–18. doi: 10.1111/add.70154 (PMC12586757; doi:10.1111/add.70154)
Supplement: Supplementary file 1 — Appendix S1: Combining data from the Health Survey for England (HSE) and Scottish Health Survey (SHeS): A technical note. [file ADD-120-2500-s001.docx]

**Appendix S1 Combining data from the Health Survey for England (HSE) and Scottish Health Survey (SHeS): A technical note.**

The analysis dataset was created by combining data from the Health Survey for England (HSE) ^[[1]](#endnote-1)^ and the Scottish Health Survey (SHeS) ^[[2]](#endnote-2)^. Multiple years were combined to maximise the sample size available for analysis.

The HSE and SHeS are both annual, large-scale, random probability surveys designed to monitor trends in their respective nation's health. In both surveys, all adults and selected children are invited to be interviewed at participating households. The main interviews cover health-related topics and demographic information and are carried out face-to-face. Respondents aged 16 years and over are additionally asked to complete a self-completion interview.

The sample designs of the two surveys are closely aligned. Both are surveys of individuals living in private households and both use the Royal Mail's Postcode Address File as a sampling frame. Addresses for each are selected using a two-stage clustered approach designed to maximise fieldwork efficiencies. During sampling, both surveys employ health geography as their first stratifier. The HSE uses Strategic Health Authority whilst SHeS uses the corresponding Scottish Health Board. These region variables were subsequently used as the stratifier variable during data analysis.

Fieldwork for both surveys is run continuously, with sampling units randomly allocated to fieldwork months to ensure an even distribution of sample throughout the year. Fieldwork is carried out by the same research agency (the National Centre for Social Research and their Scottish counterpart, the Scottish Centre for Social Research) using the same approach in terms of advance letters and minimum calling strategies.

In both surveys the main interview is conducted face-to-face, followed by paper self-completion questionnaires, and direct measurements of height and weight. In each survey the relationship information used in this analysis was collected during the main interview. The gambling and health measures were collected as part of the self-completion booklet.

Gambling questions were first introduced to both surveys in 2012, with an identical set of questions across surveys. At that time a review was conducted to assess the robustness of combining data from the two surveys, concluding it was appropriate to do so^[[3]](#endnote-3)^. More information on harmonisation of outcomes and exposures is given below.

**Data preparation**

The first step in the data preparation was to identify which survey years contained information about gambling. Both surveys are modular, with a questionnaire that combines a core set of health questions with a set of modules containing questions on specific topics that change year on year. The gambling module includes a set of questions about gambling activities, gambling frequency, and the Problem Gambling Severity Index. The combined dataset took data from HSE 2012, 2015, 2016, and 2018, and SHeS 2012, 2015, 2016 and 2017, where this module was present.

Data for both surveys was accessed via the UK Data Service. The analysis required spouses and cohabiting couples to be identified in the data. To do this, the HSE data for 2015, 2016 and 2018 were obtained under Special Licence from the UK Data Service to enable household identifiers to be accessed. The HSE 2012 and SHeS datasets already include household indicators as part of their standard access arrangements. In addition to household identifiers, the data included information on the relationships of each household member to all others.

These data were combined (each year of data appended) to create a single dataset of individuals aged 16 years or over that covered both England and Scotland.

The dataset excluded any information collected as part of any child boosts surveys (where data is only collected from children in a selected household). SHeS includes a Health Board boost sample each year, to increase the number of interviews in smaller Health Boards. These boosts have been included and the data weighted accordingly. The combined data set was weighted to be representative of adults 16 years and over living in England and Scotland, more information about these weights is given below.

**Dataset creation**

The analysis used the Problem Gambling Screening Index (PGSI) to measure gambling harms. This is a validated tool of nine questions that was included in each survey as part of the self-completion booklet. The self-completion booklet also included questions on gambling participation, with the same question and response options included in each survey.

In addition to the gambling modules, the analysis required a set of health measures to be used as outcomes, and a range of demographic variables to act as control variables.

A set of relevant variables were identified in the two surveys. These were reviewed to ensure they were available in the relevant survey years and were either compatible or could be made compatible by recoding/combining response categories.

The health outcomes were presence of a long-term (12-months) mental health condition, the 14-item Warwick-Edinburgh Mental Wellbeing Scale (WEMWBS), the 12-item General Health Questionnaire (GHQ-12), and a scale question measuring life satisfaction. WEMWBS and GHQ-12 are both validated scales calculated from standard tools that were applied in the same manner in each survey. The questions wording for life-satisfaction was also identically asked in each survey. Presence of a long-term mental health condition was collected as part of the core questionnaire, again, using the same question wording in each survey. This question was included in every survey year. WEMWBS, GHQ-12 and life satisfaction were included as part of the self-completion modules on emotional wellbeing. As such, they are not available for every year. WEMWBS is missing for HSE 2018, GHQ-12 was not included on HSE 2015, and the life satisfaction score was not included in HSE 2012 or 2015.

Demographics include sex, age (grouped into ten-year bands), ethnicity (coded as white, other due to small base sizes), religion (coded as no religion, Christian, Other), the individual’s weekly alcohol consumption (non-drinker; moderate (men < 22 units /women < 15 units); hazardous (men 22-50 units /women 15-35 units); harmful (men 50+ units/ women 35+ units)), smoking status (never; ex-smoker (occasional); ex-smoker (regular); current smoker), passive smoking exposure (yes/no), the individual’s economic activity (full/part time employment, education, or training), their highest educational qualification (Degree or higher; A-levels or equivalent; GCSE or equivalent, other, none), their National Statistics-Socio-Economic Classification (NS-SEC, 5 groups), equivalized household income (5 groups based on quintiles), tenure, number of cars in the household, whether the couple were married or cohabiting, and household type (based on household size and presence of children aged 0 to 15 years in the household).

Local deprivation was measured using English and Scottish Indices of Multiple Deprivation (IMD) scores matched at the ‘Output Area’ and quintiled for analysis, within each survey year the most recent version of IMD was used, meaning the quintile identifies the deprivation quintile for a specific survey year and country. Urbanicity was measures using the ONS urban-rural classification and grouped into urban / other. Finally, region of residence was based on government Region, with Scotland included as a separate Region. These were available in both surveys and all relevant years.

Once the relevant variables were identified and made comparable, the datasets were appended (added together) to create a single dataset. This file included identifiers for survey source (HSE/SHeS) and survey year.

**Weighting**

Non-response weighting was used in both surveys to reduce the impact of non-response bias in both surveys. The weighting schemes employed by each survey are very similar: a weight for members of participating households is generated by calibrating an initial selection weight to population estimates of age/sex and region, with the same age-bands used in each survey. A further weight for analysis of data collected as part of the main interview is generated by modelling individual response behaviour using a logistic regression with individual response as the outcome variable and a set of covariates that includes age/sex, region, a measure of household type/household size, and a measure of employment/social class of the household representative person. Full details of the weighting strategies used for the HSE and SHeS individually can be found in their respective technical reports, available via the websites above.

Three additional sets of weights were created for the combined data:

- **A new interview weight**. This was generated by scaling the existing interview weights from each survey so that the weighted distribution of the combined data matched the combined population distribution of England and Scotland (newintwt).
- **A gambling participation weight.** This was generated separately for each survey year by weighting the individuals who had responded to the participation questions in the gambling module to their corresponding population profiles of age-by-gender and region (Region for the HSE, Health Board for the SHeS), taken from their respective mid-year population estimates. The weight was generated using calibration weighting methods with the interview weight used as a starting weight. The inclusion of the interview weight means the calibrated gambling participation weights incorporate the properties of the interview weight, hence the final weights address selection bias, health board sample boosts (in SHeS) and non-response to the main interview, in addition to non-response to the gambling participation questions. The subsequent weights for each survey year were then combined and re-scaled so that the final weight is representative of the combined profile of England and Scotland. This weight is intended for any analyses of the gambling participation questions (wt_pg).
- **A PGSI participation weight** that weighted the data for non-response to the PGSI screen. The approach taken is similar to that used to generate the gambling participation weight. PGSI participation weights were generated separately for each survey year by weighting the individuals who had responded to the PGSI screen (combined with those who did not gamble and were given a PGSI score of zero) to their respective population profiles. The weight was generated using calibration weighting methods with the interview weight used as a starting weight. The subsequent weights for each survey year were then combined and re-scaled so that the final weight is representative of the combined profile of England and Scotland. This weight is intended for any analyses that incorporate the PGSI (wt_pgsi).

**Cluster and strata variables**

The analysis was carried out in Stata 18 using the ‘svy’ suite of commands. In addition to weighting, these commands allow the primary sample units (clusters) and stratification variables used during sampling to be identified. This information is used to calculate accurate standard errors. Unique (anonymised) household identifiers were used as the clustering variable, to account for clustering of cohabiting couples within households. The stratification variable was the regional stratifier used as the main stratifier during sample selection (Strategic Health Area/Region in HSE, Scottish Health Board in SHeS), split by year.

1. <https://digital.nhs.uk/data-and-information/publications/statistical/health-survey-for-england> [↑](#endnote-ref-1)
2. <https://www.gov.scot/collections/scottish-health-survey> [↑](#endnote-ref-2)
3. Wardle H. et al (2014) Gambling behaviour in England and Scotland: Findings from the Health Survey for England 2012 and Scottish Health Survey 2012. Report prepared for the Gambling Commission. <https://ucalgary.scholaris.ca/server/api/core/bitstreams/491f55f9-61e9-4185-8a1f-c6d402675403/content> [↑](#endnote-ref-3)
