# Supplementary material for: Exploring the impact on emotional wellbeing of having a spouse or cohabiting partner with elevated Problem Gambling Severity Index (PGSI) scores: Secondary analysis of cross‐sectional health survey data
Source: Addiction. 2025 Sep 3;120(12):2500–18. doi: 10.1111/add.70154 (PMC12586757; doi:10.1111/add.70154)
Supplement: Supplementary file 2 — Appendix S2: Treatment of missing values. [file ADD-120-2500-s002.docx]

**Appendix S2 Treatment of missing values**

This document sets out the number of missing values for each variable included in the analysis and the treatment applied to those values within the modelling. Analysis is based on 20,091 individuals who were in a cohabiting couple, whose own PGSI score was equal to zero, and where there was information available on their partner’s PGSI.

| **Type** | **Variable label** | **Missing** | **Treatment** |
| --- | --- | --- | --- |
| Outcome | Long-term mental health disorder | 9 | Excluded |
| Outcome | Overall satisfaction with life nowadays | 6,600 | Excluded |
| Outcome | WEMWBS score | 4,710 | Excluded |
| Outcome | General health bad or very bad | 0 |  |
| Outcome | GHQ-12 score using likert coding | 3,568 | Excluded |
| Exposure | PGSI score | 0 | NA |
| Exposure | Spouse PGSI score | 0 | NA |
| Control | Survey year | 0 |  |
| Control | Sex | 0 |  |
| Control | Age group | 0 |  |
| Control | Ethnicity | 18 | White |
| Control | Religion | 144 | Christian |
| Control | Marital status | 0 | NA |
| Control | Number of cars normally available | 2 | One car |
| Control | Tenure recoded | 24 | Outright owner |
| Control | Equivalised Income Quintiles | 2,514 | Own category |
| Control | Highest educational qualification | 13 | No quals |
| Control | Economic activity condensed | 20 | Other inactive |
| Control | Individual NS-SEC classification | 385 | Own category |
| Control | Whether spent money on any gambling activity in last 12 months | 0 | NA |
| Control | weekly drinking category | 166 | Moderate drinker |
| Control | Cigarette Smoking Status | 8 | Never smoked |
| Control | Ever exposed to passive smoke in own or others home | 8 | Never exposed |
| Control | Ever had high blood pressure (also known as hypertension) | 22 | No high blood pressure |
| Control | Ever had diabetes | 5 | No diabetes |
| Control | Limiting long-lasting illness | 10 | No limiting illness |
| Control | Government Office Region | 0 | NA |
| Control | Rurality of dwelling unit (urban/rural) | 4 | Urban |
| Control | Quintiles of IMD score (based on survey year/country) | 0 | NA |
| Control | Household type | 2 | Small adult household |
