# Supplementary material for: Exploring the impact on emotional wellbeing of having a spouse or cohabiting partner with elevated Problem Gambling Severity Index (PGSI) scores: Secondary analysis of cross‐sectional health survey data
Source: Addiction. 2025 Sep 3;120(12):2500–18. doi: 10.1111/add.70154 (PMC12586757; doi:10.1111/add.70154)
Supplement: Supplementary file 3 — Appendix S3: Supplementary results from Poisson analysis of GHQ‐12 Table S3.1: Unadjusted and adjusted regression output: GHQ‐12. [file ADD-120-2500-s003.docx]

**Appendix S3 – Supplementary results from Poisson analysis of GHQ-12**

The diagnostics for the GHQ-12 linear regression model suggested the normality assumption did not fully hold. A different approach to the modelling may have been more suitable. To test this, the GHQ-12 analysis was repeated using Poisson regression and using the original, unstandardised GHQ-12 Likert scale as the outcome variable. Poisson regression was selected owing to the right-skewed non-negative distribution of the GHQ-12 Likert scale. Whilst Poisson regression is designed for count data, the Poisson estimates are fully robust for the mean parameters when robust standard errors are applied^[[1]](#endnote-1)^.

As with the linear regression, the analysis used the svy commands in Stata V18 to account for clustering and weights and produce appropriate standard errors. The same set of control variables were used in the adjusted model. Both unadjusted and adjusted regression models show statistically significant associations between the full range of partner’s PGSI and GHQ-12 (Adjusted model p-value: 0.043, IRR. 1.008; 95% CI: 1.000, 1.016), corroborating the associations indicated by the linear regression model.

**Table S3.1: Unadjusted and adjusted regression output: GHQ-12**

| **Baseline** | **Categories** | **IRR** | **95% CI** | **z-test** | **P-value** |
| --- | --- | --- | --- | --- | --- |
|  |  |  |  |  |  |
| **Unadjusted** |  |  |  |  |  |
| Partner PGSI score |  | 1.015 | 1.007,1.024 | 3.6 | 0.000 |
| Constant |  | 10.641 | 10.543,10.740 | 502.2 | 0.000 |
|  |  |  |  |  |  |
| **Adjusted** |  |  |  |  |  |
| Partner PGSI score |  | 1.008 | 1.000,1.016 | 2.0 | 0.043 |
| Individual had not spent money on any gambling activity in last 12 months | Individual had spent money on any gambling activity in last 12 months | 0.998 | 0.981,1.015 | -0.3 | 0.791 |
| 16-24 | 25-34 | 1.014 | 0.958,1.074 | 0.5 | 0.622 |
|  | 35-44 | 1.071 | 1.011,1.136 | 2.3 | 0.021 |
|  | 45-54 | 1.050 | 0.989,1.115 | 1.6 | 0.111 |
|  | 55-64 | 1.013 | 0.952,1.078 | 0.4 | 0.678 |
|  | 65-74 | 0.981 | 0.909,1.059 | -0.5 | 0.627 |
|  | 75+ | 0.988 | 0.909,1.073 | -0.3 | 0.775 |
| Male | Female | 1.054 | 1.037,1.072 | 6.4 | 0.000 |
| Other | White | 1.092 | 1.043,1.143 | 3.8 | 0.000 |
| No religion | Christian - Catholic | 0.985 | 0.961,1.009 | -1.2 | 0.219 |
|  | Christian all other denominations | 0.998 | 0.979,1.017 | -0.2 | 0.837 |
| In employment, self emp or govt training | Any other religion | 1.048 | 0.994,1.105 | 1.8 | 0.080 |
|  | In full-time education | 1.094 | 1.010,1.185 | 2.2 | 0.028 |
|  | Retired | 0.948 | 0.918,0.980 | -3.2 | 0.001 |
|  | ILO unemployed | 1.144 | 1.067,1.226 | 3.8 | 0.000 |
|  | Other inactive | 1.136 | 1.101,1.172 | 8.1 | 0.000 |
| Degree (or equivalent) or higher | Higher education below degree | 0.992 | 0.966,1.019 | -0.6 | 0.574 |
|  | A-level / Scottish highers / or equivalent | 0.980 | 0.954,1.007 | -1.4 | 0.150 |
|  | GCSE /Scottish Standard Grades / or equivalent | 0.991 | 0.966,1.017 | -0.7 | 0.502 |
|  | Other | 0.946 | 0.879,1.018 | -1.5 | 0.139 |
|  | No qualifications | 1.004 | 0.971,1.037 | 0.2 | 0.829 |
| Missing | Managerial and professional occupations | 1.067 | 0.992,1.147 | 1.7 | 0.083 |
|  | Intermediate occupations | 1.082 | 1.004,1.166 | 2.1 | 0.038 |
|  | Small employers and own account workers | 1.064 | 0.985,1.149 | 1.6 | 0.113 |
|  | Lower supervisory and technical occupations | 1.045 | 0.968,1.129 | 1.1 | 0.262 |
|  | Semi-routine occupations | 1.045 | 0.971,1.124 | 1.2 | 0.238 |
| Missing | Lowest Quintile (<=£14,918) | 1.016 | 0.984,1.048 | 1.0 | 0.331 |
|  | Second lowest Quintile (>£14,918 <= £23,084) | 1.020 | 0.989,1.052 | 1.3 | 0.204 |
|  | Middle Quintile (>£23,084 <=£31,967) | 1.017 | 0.986,1.049 | 1.1 | 0.291 |
|  | Second highest Quintile (>£31,967 <=£52,817) | 1.021 | 0.988,1.056 | 1.2 | 0.215 |
|  | Highest Quintile (>£52,817) | 1.079 | 1.040,1.120 | 4.0 | 0.000 |
| Buying with mortgage/loan | Own it outright | 0.986 | 0.967,1.005 | -1.4 | 0.149 |
|  | Part rent/part mortgage | 1.044 | 0.946,1.152 | 0.9 | 0.394 |
|  | Rent (including rents paid by housing benefit) | 0.981 | 0.956,1.007 | -1.4 | 0.158 |
|  | Living here rent free | 0.995 | 0.908,1.090 | -0.1 | 0.912 |
| None | One | 1.008 | 0.973,1.045 | 0.4 | 0.658 |
|  | Two | 0.991 | 0.954,1.028 | -0.5 | 0.624 |
|  | Three or more | 0.995 | 0.948,1.044 | -0.2 | 0.844 |
| Non-drinker | moderate (men up to and including 21 /women up to and including 14) | 0.967 | 0.939,0.996 | -2.3 | 0.024 |
|  | hazardous (men over 21 up to and including 50/women over 14 up to and including | 0.963 | 0.931,0.997 | -2.1 | 0.034 |
|  | harmful (men over 50/ women over 35) | 0.998 | 0.951,1.049 | -0.1 | 0.947 |
| Never smoked cigarettes at all | Used to smoke cigarettes occasionally | 1.049 | 1.013,1.087 | 2.7 | 0.008 |
|  | Used to smoke cigarettes regularly | 1.004 | 0.986,1.023 | 0.4 | 0.653 |
|  | Current cigarette smoker | 1.009 | 0.981,1.036 | 0.6 | 0.542 |
| Never exposed | Exposed | 1.037 | 1.008,1.066 | 2.5 | 0.011 |
| Yes | No | 0.963 | 0.944,0.983 | -3.7 | 0.000 |
| Yes | No | 0.976 | 0.942,1.012 | -1.3 | 0.185 |
| Limiting long-lasting illness | Non-limiting long-lasting illness | 0.772 | 0.754,0.792 | -20.6 | 0.000 |
|  | No limiting long-lasting illness | 0.738 | 0.722,0.754 | -27.9 | 0.000 |
| Married / civil partnership | Living as married | 1.026 | 1.000,1.052 | 2.0 | 0.046 |
| Small family: two adults of any age and one or two children | Older smaller family: 1 adult under 65 and one adult 65+, or two adults 65+ and | 0.976 | 0.931,1.023 | -1.0 | 0.308 |
|  | Large adult: 3+ adults, no children | 1.021 | 0.987,1.056 | 1.2 | 0.233 |
|  | Small adult: 2 adults under 65 and no children | 1.018 | 0.992,1.046 | 1.3 | 0.178 |
|  | Large family: 2 adults of any age and 3+ children or 3+ adults and 1+ children | 1.025 | 0.992,1.059 | 1.5 | 0.134 |
| Least deprived | 2nd | 1.018 | 0.995,1.041 | 1.5 | 0.129 |
|  | 3rd | 1.012 | 0.988,1.038 | 1.0 | 0.330 |
|  | 4th | 1.019 | 0.991,1.047 | 1.3 | 0.181 |
|  | Most deprived | 0.999 | 0.966,1.032 | -0.1 | 0.940 |
| Urban | Town/ Fringe/ Village, hamlet and isolated dwellings | 0.994 | 0.974,1.014 | -0.6 | 0.539 |
| North East | North West | 0.974 | 0.933,1.017 | -1.2 | 0.229 |
|  | Yorkshire and the Humber | 1.008 | 0.963,1.056 | 0.4 | 0.720 |
|  | East Midlands | 1.012 | 0.968,1.059 | 0.5 | 0.586 |
|  | West Midlands | 1.019 | 0.974,1.066 | 0.8 | 0.415 |
|  | East of England | 0.998 | 0.956,1.043 | -0.1 | 0.937 |
|  | London | 0.980 | 0.933,1.029 | -0.8 | 0.415 |
|  | South East | 1.002 | 0.962,1.045 | 0.1 | 0.910 |
|  | South West | 0.997 | 0.956,1.040 | -0.1 | 0.898 |
|  | Scotland | 0.951 | 0.916,0.988 | -2.6 | 0.010 |
| General health very good/good | Fair |  |  |  |  |
|  | Bad/Very bad |  |  |  |  |
| Survey year (2012) | 2015 | 1.013 | 0.983,1.044 | 0.8 | 0.397 |
|  | 2016 | 1.03 | 1.009,1.052 | 2.8 | 0.006 |
|  | 2017/8 | 1.001 | 0.980,1.023 | 0.1 | 0.900 |
|  |  |  |  |  |  |
| Constant |  | 11.384 | 10.053,12.891 | 38.3 | 0.000 |
|  |  |  |  |  |  |
| Base (unweighted) |  | 16521 |  |  |  |

1. Wooldridge J. (2010) Econometric Analysis of Cross Section and Panel Data, second edition. Cambridge, MA: MIT Press. [↑](#endnote-ref-1)
