# Supplementary material for: Exploring the impact on emotional wellbeing of having a spouse or cohabiting partner with elevated Problem Gambling Severity Index (PGSI) scores: Secondary analysis of cross‐sectional health survey data
Source: Addiction. 2025 Sep 3;120(12):2500–18. doi: 10.1111/add.70154 (PMC12586757; doi:10.1111/add.70154)
Supplement: Supplementary file 4 — Appendix S4: E‐values for Partner PGSI. Table S4.1: E‐values for partner PGSI (continuous outcomes). Table S4.2: E‐values for partner PGSI (binary outcome). [file ADD-120-2500-s004.docx]

**Appendix S4: E-values for Partner PGSI**

E-values were proposed by VanderWeele and Ding^[[1]](#endnote-1)^ as a means of determining the strength of existing associations against unmeasured confounders. The e-value is calculated from the coefficients or odds ratios of the exposure (partner PGSI score) as per the formula in VanderWeele and Ding’s 2017 paper. The e-values for adjusted models are shown below. The coefficients from the linear regression models were adjusted to create approximate risk ratios based on the formula in Table 2 of VanderWeele and Ding’s 2017 paper.

Table S4.1 E-values for partner PGSI (continuous outcomes)

| **Outcome** | **Coefficient for partner PGSI (adjusted model)** | **95% CI for the partner PGSI coefficient (adjusted model)** | **E-value for the coefficient** | **E-value for the lower limit of the 95% CI** |
| --- | --- | --- | --- | --- |
| WEMWBS | 0.022 | 0.004,0.040 | 1.164 | 1.064 |
| GHQ-12 | 0.021 | 0.000,0.043 | 1.160 | 1.012 |
| Life Sat | 0.036 | 0.005,0.067 | 1.219 | 1.073 |

Table S4.2 E-values for partner PGSI (binary outcome)

| **Outcome** | **Odds Ratio for partner PGSI (adjusted model)** | **95% CI for the partner PGSI OR (adjusted model)** | **E-value for the OR** | **E-value for the lower limit of the 95% CI** |
| --- | --- | --- | --- | --- |
| Long-term mental health condition | 1.023 | 0.965,1.086 | 1.176 | 1.000 |

1. VanderWeele TJ, Ding P. Sensitivity Analysis in Observational Research: Introducing the E-Value. Ann Intern Med. 2017 Aug 15;167(4):268-274. doi: 10.7326/M16-2607. Epub 2017 Jul 11. PMID: 28693043. [↑](#endnote-ref-1)
