# Supplementary material for: Exploring the impact on emotional wellbeing of having a spouse or cohabiting partner with elevated Problem Gambling Severity Index (PGSI) scores: Secondary analysis of cross‐sectional health survey data
Source: Addiction. 2025 Sep 3;120(12):2500–18. doi: 10.1111/add.70154 (PMC12586757; doi:10.1111/add.70154)
Supplement: Supplementary file 5 — Appendix S5: STROBE Statement—Checklist of items that should be included in reports of cohort studies. [file ADD-120-2500-s005.docx]

**Appendix S5:** STROBE Statement—Checklist of items that should be included in reports of ***cohort studies***

|  | Item No | Recommendation |
| --- | --- | --- |
| **Title and abstract** | 1 | Indicate the study’s design with a commonly used term in the title or the abstract  *Done – title is “**Impact of an individual’s elevated PGSI scores on the emotional wellbeing of their spouse or cohabiting partner: a secondary analysis of health data”* |
|  |  | Provide in the abstract an informative and balanced summary of what was done and what was found  *Completed – see Abstract* |
| Introduction | | |
| Background/rationale | 2 | Explain the scientific background and rationale for the investigation being reported  *See Introduction, paragraphs 1-4* |
| Objectives | 3 | State specific objectives, including any prespecified hypotheses  *See Introduction, paragraph 5* |
| Methods | | |
| Study design | 4 | Present key elements of study design early in the paper  *See Methods>Design, paragraphs 1& 2* |
| Setting | 5 | Describe the setting, locations, and relevant dates, including periods of recruitment, exposure, follow-up, and data collection  *See Methods>Design, paragraphs 1& 2* |
| Participants | 6 | Give the eligibility criteria, and the sources and methods of selection of participants. Describe methods of follow-up  *See Methods>Design, paragraph 3* |
|  |  | For matched studies, give matching criteria and number of exposed and unexposed  *Not applicable* |
| Variables | 7 | Clearly define all outcomes, exposures, predictors, potential confounders, and effect modifiers. Give diagnostic criteria, if applicable  *See Methods>Measures, paragraphs on outcome, exposures and controls* |
| Data sources/ measurement | 8* | For each variable of interest, give sources of data and details of methods of assessment (measurement). Describe comparability of assessment methods if there is more than one group  *See Methods>Measures, paragraphs on outcome, exposures and controls* |
| Bias | 9 | Describe any efforts to address potential sources of bias  *See Appendix B section on weighting* |
| Study size | 10 | Explain how the study size was arrived at  *See Methods>Design, paragraph 3.* |
| Quantitative variables | 11 | Explain how quantitative variables were handled in the analyses. If applicable, describe which groupings were chosen and why  *See Methods>Measures – sections on outcomes, exposures and controls* |
| Statistical methods | 12 | Describe all statistical methods, including those used to control for confounding  *See Methods>Analyses which describes this in detail* |
|  |  | Describe any methods used to examine subgroups and interactions  *Not applicable* |
|  |  | Explain how missing data were addressed  *See Methods>measures – for treatment of missing data for individual variables and Appendix A for fuller details* |
|  |  | If applicable, explain how loss to follow-up was addressed  *See Methods>Analysis, paragraph 1 states weights were used. Detail on weighting is provided in Appendix B* |
|  |  | Describe any sensitivity analyses  *Not applicable* |
| Results | | |
| Participants | 13* | Report numbers of individuals at each stage of study—eg numbers potentially eligible, examined for eligibility, confirmed eligible, included in the study, completing follow-up, and analysed  *See Table 1 and Methods>Design* |
|  |  | Give reasons for non-participation at each stage  *Not applicable as reasons unknown* |
|  |  | Consider use of a flow diagram  *Not applicable – cross-sectional secondary data were used* |
| Descriptive data | 14* | Give characteristics of study participants (eg demographic, clinical, social) and information on exposures and potential confounders  *See Tables 1 and 2* |
|  |  | Indicate number of participants with missing data for each variable of interest  *See Appendix A* |
|  |  | Summarise follow-up time (eg, average and total amount)  *Not applicable* |
| Outcome data | 15* | Report numbers of outcome events or summary measures over time  *See Table 1* |
| Main results | 16 | Give unadjusted estimates and, if applicable, confounder-adjusted estimates and their precision (eg, 95% confidence interval). Make clear which confounders were adjusted for and why they were included  *See Methods>Measures>Controls for list of adjustments. All models report 95% CI. Unadjusted and adjusted estimates are presented in Table 3* |
|  |  | Report category boundaries when continuous variables were categorized  *See Methods>Measures, section on exposures* |
|  |  | If relevant, consider translating estimates of relative risk into absolute risk for a meaningful time period  *Not applicable* |
| Other analyses | 17 | Report other analyses done—eg analyses of subgroups and interactions, and sensitivity analyses  *Not applicable* |
| Discussion | | |
| Key results | 18 | Summarise key results with reference to study objectives  *See Discussion, paragraph 1* |
| Limitations | 19 | Discuss limitations of the study, taking into account sources of potential bias or imprecision. Discuss both direction and magnitude of any potential bias  *See Discussion, paragraph 5* |
| Interpretation | 20 | Give a cautious overall interpretation of results considering objectives, limitations, multiplicity of analyses, results from similar studies, and other relevant evidence  *See overall discussion* |
| Generalisability | 21 | Discuss the generalisability (external validity) of the study results  *See Discussion, paragraph 4* |
| Other information | | |
| Funding | 22 | Give the source of funding and the role of the funders for the present study and, if applicable, for the original study on which the present article is based  *A statement is included on the title page* |

*Give information separately for exposed and unexposed groups.

**Note:** An Explanation and Elaboration article discusses each checklist item and gives methodological background and published examples of transparent reporting. The STROBE checklist is best used in conjunction with this article (freely available on the Web sites of PLoS Medicine at http://www.plosmedicine.org/, Annals of Internal Medicine at http://www.annals.org/, and Epidemiology at http://www.epidem.com/). Information on the STROBE Initiative is available at http://www.strobe-statement.org.
